# Chloroma

**Published:** 1916-10

**Authors:** 


**Chloroma.** E. W. Gould & L. T. Le Wald, of N. Y., Arch. of Paediatrics, June. This disease has been known since the beginning of the nineteenth century as chloroma, chlorolymphoma and green cancer. It is rare, the literature having recently been reviewed by Dock and Warthin. It occurs mainly in children and is more frequent in males. It is due to a hyperplasia of the parent cells of the leucocytes, especially in the red marrow but also in other leucoblastic tissues, so that neoplastic formations are found in the spleen, liver, lymph glands, etc. Exophthalmos, due to retro-bulbar lymphoma, involvement of the cranial bones, with swelling especially in the temporal and occipital regions, enlargement of the spleen, various lymph nodes, especially the cervical, severe, progressive, fatal anaemia, with rise of temperature, general disturbance of nutrition, especially emaciation, are the principal symptoms. The red cells are diminished by reason of lack of production and the white cells are usually increased at least at times, so that the disease has been classified as leucocythaemia. However, as in the present cases, the hyperplasia of the parent cells may reach such a stage that no excess of white cells reaches the blood—aleukaemic chloroma. The authors report cases in a boy of 5 and a girl of $3\frac{1}{2}$, with typic clinical and pathologic findings, except leukaemia and barring the absence of post mortems which were refused. X-ray examination showed separation of sutures, especially coronary and parietal and mottling of long bones, due to rarefaction of medulla, also separation of periosteum in places.

